# Cell-specific epigenetic changes in atherosclerosis

**DOI:** 10.1042/CS20201066

**Published:** 2021-05-14

**Authors:** Abdul Waheed Khan, Francesco Paneni, Karin A.M. Jandeleit-Dahm

**Affiliations:** 1 Department of Diabetes, Central Clinical School, Monash University, Melbourne, Australia; 2 Cardiovascular Epigenetics and Regenerative Medicine, Centre for Molecular Cardiology, University of Zurich, Switzerland; 3 German Diabetes Centre, Leibniz Centre for Diabetes Research at the Heinrich Heine University, Dusseldorf, Germany

**Keywords:** atherosclerosis, cardiovascular disease, Epigenetics

## Abstract

Atherosclerosis is a disease of large and medium arteries that can lead to life-threatening cerebrovascular and cardiovascular consequences such as heart failure and stroke and is a major contributor to cardiovascular-related mortality worldwide. Atherosclerosis development is a complex process that involves specific structural, functional and transcriptional changes in different vascular cell populations at different stages of the disease. The application of single-cell RNA sequencing (scRNA-seq) analysis has discovered not only disease-related cell-specific transcriptomic profiles but also novel subpopulations of cells once thought as homogenous cell populations. Vascular cells undergo specific transcriptional changes during the entire course of the disease. Epigenetics is the instruction-set-architecture in living cells that defines and maintains the cellular identity by regulating the cellular transcriptome. Although different cells contain the same genetic material, they have different epigenomic signatures. The epigenome is plastic, dynamic and highly responsive to environmental stimuli. Modifications to the epigenome are driven by an array of epigenetic enzymes generally referred to as writers, erasers and readers that define cellular fate and destiny. The reversibility of these modifications raises hope for finding novel therapeutic targets for modifiable pathological conditions including atherosclerosis where the involvement of epigenetics is increasingly appreciated. This article provides a critical review of the up-to-date research in the field of epigenetics mainly focusing on *in vivo* settings in the context of the cellular role of individual vascular cell types in the development of atherosclerosis.

## Introduction

**Figure 1:**
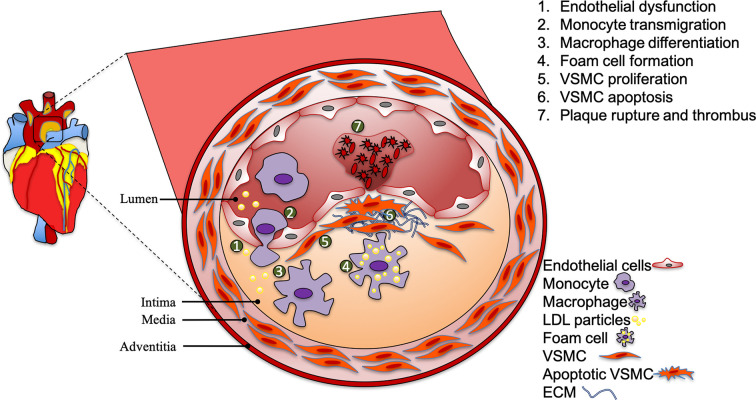
Atherosclerotic plaque development Atherosclerotic plaques usually develop at the atheroprone regions of medium and large arteries. The process is initiated by endothelial dysfunction and the retention of LDL cholesterol. This causes monocyte transmigration into the intima and differentiation into macrophages. Continuous engulfment of LDL particles by macrophages causes foam cell formation. VSMCs proliferation is thought to initially stabilize the plaque, however VSMC apoptosis contributes to plaque destabilization and cause plaque rupture. Abbreviations: ECM, extracellular matrix; LDL, low-density lipoprotein.

Atherosclerosis is characterized by a pathological build-up of plaque within the arterial vessel walls and is a major contributor to cardiovascular disease [1]. Atherosclerosis is a progressive disease where lesion progress from fatty streak to vulnerable plaques that on rupture, cause thrombosis and arterial blockage [2,3]. The interrupted blood supply to vital organs such as brain and heart caused by thrombosis leads to serious life-threatening conditions such as stroke and heart attacks [4]. Several independent risk factors including flow-mediated hemodynamic forces, hyperglycemia, hyperlipidemia, smoking and alcohol consumption have been identified in pathological plaque development [5]. The arterial wall consists of different residing and infiltrating cellular populations that undergo specific functional changes during the course of the disease development. Recent high-dimensional single-cell studies have discovered the tremendous diversity in the transcriptional profile of cells of the vascular wall. Atherosclerotic lesion formation is a complex process that involves several mechanisms including endothelial dysfunction, lipoprotein retention, inflammatory cell recruitment, oxidative stress, foam cell formation, apoptosis and necrosis, vascular smooth muscle cell (VSMC) proliferation, matrix synthesis, calcification, angiogenesis and fibrous cap formation (Figure 1) [6]. Transcriptional changes drive functional changes in all different types of vascular cells during the distinct pathological stages in atherosclerosis progression. Epigenetics controls the transcriptomic changes through enzyme-mediated epigenetic mechanisms namely histone modification and DNA methylation [7]. Epigenetic mechanisms respond to environmental stimuli in regulating the transcriptional landscape of each vascular cell type and affecting their functionality. In recent years, the field of epigenetics has gained attention among vascular clinicians and researchers [8]. In this review article, we have gathered the latest research advances in the field of epigenetics in the cellular context of atherosclerosis.

## Atherosclerotic plaque development: the role of vascular cells

The arterial wall is composed of different cell populations layered into intima, media, adventitia and perivascular adipose tissue (PVAT). Vascular structure and function is supported by the location of the vascular cells into these layers. The innermost layer of the artery, the intima is a thin and a continuous monolayer of endothelial cells that are the first cell population exposed to metabolic imbalances in the form of hyperglycemia and hyperlipidemia and to the shear stress of the blood flow [9–11]. Furthermore, the endothelium is the first barrier between blood and the rest of the vessel wall. The healthy endothelium functions in multiple ways for vascular homeostasis [6]. Endothelial dysfunction is generally referred to as abnormalities in the endothelial-derived nitric oxide bioavailability resulting in deleterious changes in vascular reactivity [6]. Impaired endothelial function is the widely accepted the initiating step in atherosclerotic plaque development. Over time, circulatory stimuli such as hyperglycemia, hyperlipidemia, hypertension and turbulent blood flow (TBF) compromise endothelial function. Cholesterol carrying low-density lipoprotein (LDL) is transported transcellularly through the endothelial cells via transcytosis into subendothelial space where it is modified through biochemical processes into oxidized LDL [6,12]. The retention of oxLDL at sites of TBF stimulates endothelial cells to express adhesion molecules such as vascular cell adhesion molecule 1 (VCAM1) and produce pro-inflammatory cytokines that initiate an inflammatory cascade with immunocytes recruitment into subendothelial space [13]. Leukocytes cross-endothelial migration initiates the process of monocyte differentiation into macrophages and continuous engulfment of oxLDL by macrophages over time leads to foam cell formation [13]. VSMCs along with elastin and extracellular collagen form the media of the vascular wall. VSMCs’ migration, proliferation and de-differentiation initially stabilize the atherosclerotic plaque by forming the fibrous cap through production of extracellular matrix proteins such as collagen [14]. Usually considered beneficial, recent evidence suggests a more complex role for VSMCs with oligoclonal expansion and transdifferentiation into macrophages and mesenchymal stem cell-like cells which can also contribute to lesion instability [15]. The external layer of the vessel wall is the adventitia that consists of fibroblasts, collagen, elastic fibers, vaso vasorum, adrenergic nerves and lymphatic vessels. In response to vascular injury, fibroblasts proliferate and migrate to the intima where they secrete factors to regulate the growth of endothelial cells and VSMC and recruit inflammatory cells [16]. However, recent evidence suggests that fibroblasts are highly plastic and heterogeneous [17]. Blood vessels are surrounded by PVAT and emerging evidence suggest that the PVAT plays an important role in vascular contractility and inflammation but also releases adiponectin which has anti-inflammatory, insulin-sensitizing and vasodilatory properties. Positive or negative roles of different vascular cells at different stages of atherosclerosis indicate that the understanding of functions of individual cell types of the vascular wall in promotion of vascular damage is important in identifying novel cell-specific therapeutic targets. With the advent of state-of-the-art technologies that can define the transcriptomic landscape at the level of single-cell resolution such as single-cell RNA sequencing (scRNA-seq) and high- dimensional protein analysis such as cytometry time of flight (CyTOF), we are at the verge of phenomenal discoveries in the area of vascular research.

## Vascular cell heterogeneity in atherosclerosis: insights from single-cell sequencing

Unbiased bulk RNA sequencing at a greater depth has discovered several dysregulated pathways and disease-specific gene expression changes associated with atherosclerosis. However, this valuable information is irrespective of cell-specific transcriptomic changes in response to vascular injury. Combining deep sequencing with Fluorescence-assisted cell sorting (FACS) has identified cell-specific gene expression changes for example in effector T cells. However, the limitation in this method is that we need to know the surface marker first to isolate that cell type from a mixture of vascular cells. Different cell populations of arteries undergo specific gene expression changes during the process of atherosclerosis development. Thus, it is of utmost importance to identify cell-specific gene expression changes during different stages of atherosclerosis development. The expansion in sequencing technologies such as scRNA-seq and in tools that uses large combination of protein markers such as CyTOF have uncovered transcriptomic and high-dimensional protein analysis of cellular subpopulations present in atherosclerotic plaques.

Single cell sequencing revealed unexpected diversity among vascular cells. Application of single cell transcriptomic analysis in human plaques as well as in murine models of atherosclerosis has identified not only additional cell subpopulations but also cell-specific atherosclerotic transcriptomic profiles. Either the whole murine aorta or leukocytes enriched cellular mixture have been subjected to scRNA-seq to investigate the cellular heterogeneity among vascular cells. Application of scRNA-seq on FACS-sorted CD45^+^ aortic leukocytes has identified 11–13 distinct immune cell populations and their transcriptional landscape in chow and western diet-fed *Apoe^−/−^* and *Ldlr^−/−^* mice [18–20]. ScRNA sequencing data were validated by CyTOF as a second method that also identified a similar number of distinct immunocyte populations. Interestingly, these studies have revealed previously unknown heterogeneous macrophages subtypes including Trem2^hi^ macrophages. Foamy macrophages (CD45^+^SSC^hi^BODIPY^hi^) with similar cell identity as Trem2^hi^ macrophages were also identified in atherosclerotic mouse models [20]. These cells were identified as intimal lipid-loaded macrophages that lack an inflammatory phenotype with the expression of only few inflammatory genes. Instead, non-foamy macrophages were found expressing interleukin (IL)-1β and other inflammatory transcripts confirming a pro-inflammatory phenotype [20–22]. However, the analysis in these studies is limited to CD45 positive cells excluding all other vascular cells. Other studies have applied single cell sequencing approach to investigate cellular diversity and transcriptional changes in the whole healthy and diseased aorta [23,24]. These studies have identified plasticity and heterogeneity among vascular cells including endothelial, fibroblasts and VSMCs. Massive parallel sequencing at a single cell level has also been used in human plaques. Application of single cell sequencing including RNA and ATAC sequencing on advanced human carotid atherosclerotic plaque identified intercellular communication and defined their transcriptomic and epigenomic landscape [25]. Gene expression profiles of endothelial cells indicate not only an activated state but also a potential transdifferentiating state. Furthermore, scRNA-seq analysis of murine and human (carotid and coronary artery) plaques identified VSMCs transition to smooth muscle cell-derived intermediate cells that were multipotent and could differentiate into macrophage-like and fibrochondrocyte-like cells [26]. These studies not only show the complexity of gene expression changes in the interconnected vascular cells but also emphasize the plasticity and heterogeneity of these cells in the healthy and diseased vessel (Table 1). Studying epigenomics at single cell resolution represents technical challenges. Methods have been developed to study epigenome at single cell resolution including single cell DNA methylome sequencing to measure DNA methylation, single cell ATAC sequencing to measure chromatin accessibility, single cell chromatin immunoprecipitation (ChIP) sequencing to measure histone modifications. Single cell ChIP sequencing represent technical challenges due to background noise of nonspecific pulldown by antibodies and has not been broadly used. So far, studies of single cell epigenomics in atherosclerosis is limited to single cell ATAC-seq primarily due to limited number of viable cells from atherosclerotic vessels [27].

**Table 1:** Heterogeneity among vascular cell populations as defined by scRNA-seq

S.No	Health status	Species	Vascular compartment	ECs	VSMCs	Mφ	Fib	Description/Main findings
1	Healthy	mouse	Whole aorta	3	1	1	2	Most significant heterogeneity was observed in ECs. The two major EC subpopulations (*Vcam1*^+^/*Cd36*^−^ and *Vcam1*^−^/*Cd36*^+^) showed unique distribution pattern in the lesser and greater curvatures that may be the result of differences in blood flow and shear stress [24].
2	Healthy	Mouse (C57BL/6)	Endothelium of descending aorta	2	-	-	-	scRNA-seq analysis of CD34^+^ (isolated via FACs) cells revealed two distinct aortic endothelial populations. The authors suggested that the progenitor cells (*Pdgfr, Sox9, Il33, Postn, Dcn*) transition to differentiated cells [23].
3	Atherosclerotic	Mouse (*Ldlr*^−/−^)	CD45^+^ aortic cells	-	-	3	-	ScRNA-seq analysis of CD45^+^ cells identified 13 leukocytes’ populations with three major Mφ populations including two atherosclerosis-specific population that are inflammatory and foamy *Trem*2^hi^ Mφ [18].
4	Atherosclerotic	Mouse (*Apoe*^−/−^)	CD45^+^ aortic cells (thoracic and abdominal)	-	-	1	-	ScRNA-seq analysis of CD45^+^ cells identified 11 leukocytes’ populations including three B-cell subsets. The results were confirmed by CYTOF as a second method [19].
5	Atherosclerotic	Human	CD45^+^ cells of carotid artery plaques	-	-	2		Single cell proteomic (CyTOF) and transcriptomic (CITE-seq and scRNA-seq) of plaque and blood from same patients unraveled distinct feature of both T cells and macrophages in symptomatic and asymptomatic disease [182].
6	Atherosclerotic	Mouse (*Ldlr*^−/−^)	CD45^+^ aortic cells	-	-	2	-	ScRNA-seq analysis of CD45^+^ cells identified 11 leukocytes. Macrophages were the largest population. Inflammatory genes were down-regulated in foamy macrophages whereas intimal non-foamy macrophages showed a distinct inflammatory phenotype [20].
7	Atherosclerotic	human	Carotid artery plaques	2	2	3		This is the first study that also covers non-immune cells in human plaque. In total, 14 cell populations were identified with 11 leukocytes and 3 non-immune cellular clusters. Transcriptional data of endothelial subpopulations are suggestive of an activated and a transitory to mesenchymal phenotype. SMCs showed a contractile and synthetic phenotype. One B cell and four T cell clusters were also identified. Mφ included two pro-inflammatory Mφ and a foamy Trem2^hi^ Mφ populations [25].
8	Atherosclerotic	Mouse (*Ldlr*^−/−^)	CD11^+^ CX3CR1^+^ monocyte lineage aortic arch cells	-	-	3	-	ScRNA-seq analysis combined with genetic fate mapping profiled plaque cells derived from CX3CR1 precursors in plaque during progression and regression of atherosclerosis. The present study tracks the cellular state during the differentiation of CXC3R1 cells into macrophages in atherosclerosis. Eleven cellular clusters were identified including three macrophages identified by Cochain et al. confirming the heterogeneity of macrophages. They also identified a proliferating monocyte cluster with a stem-like phenotype [183].
9	Atherosclerotic	Mouse (*Apoe*^−/−^)	Aortic leukocytes	-	-	2	-	ScRNA-seq analysis of aortic leukocytes after macrophage-specific nano-therapy using single-walled carbon nanotubes (SWNT). The data revealed that pro-phagocytic SWNT decreased inflammatory phenotype in macrophages [184].
10	Atherosclerotic	Mouse (*Ldlr^−/-,^ Apoe*^−/−^) and human	Mouse (Ascending and thoracic aorta and brachiocephalic artery) and human (carotid plaques)	2	3	3	2	ScRNA-seq in SMC-lineage tracing mice identified multiple SMC-derived cell state during atherosclerosis. SMC may transition through an intermediate cell state (termed as SEM cells by the authors) to multiple cell types [26].
11	Healthy and atherosclerotic	Mouse (C57BL/6and *Apoe*^−/−^)	medial cells aortic arch and thoracic aorta	-	3	-	-	ScRNA-seq combined with SMC-lineage tracing identified a rare population of multipotent progenitor marker Sca1+ VSMCs which was shown to be a hallmark of VSMC transition from contractile to inflammatory phenotype [107].
12	Atherosclerotic	Mouse (*Apoe*^−/−^) and human	Aortic root in mice and right coronary artery in human	3(m) 1(h)	3(m) 2(h)	1(m) 1(h)	2(m) 2(h)	scRNA-seq combined with SMC-lineage tracing identified a transition of SMC to fibroblast-like cells which were also present in human plaques [108].
13	Atherosclerotic	Mouse (*Apoe*^−/−^)	Adventitia of the whole aorta	1	1	2	4	This scRNA-seq atlas of aortic adventitia characterized resident and bone marrow-derived cell populations with identification of mesenchyme cells expression stem/progenitor markers that could be a source to several differentiated cells [131].

Abbreviations: EC, endothelial cell; Fib’, fibroblasts; h, human; m, mouse; Mφ, macrophage.

## Epigenetics

**Figure 2:**
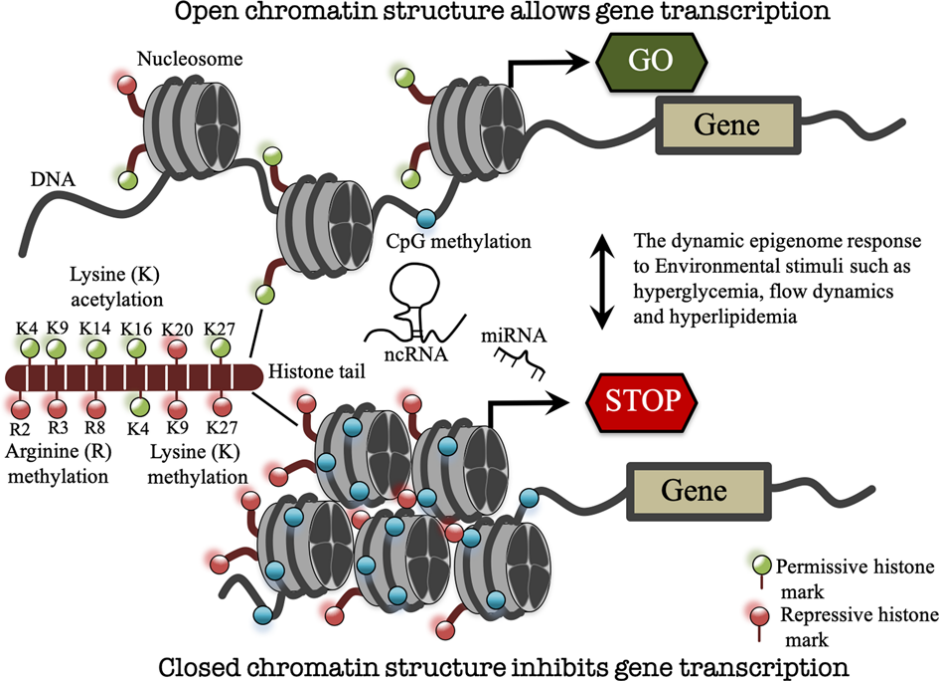
The dynamic epigenome In response to environmental cues, genes can be turned on and off by reversible epigenetic changes. Epigenetic mechanisms namely DNA methylation and histone modifications (such as arginine (R) methylation, lysine (K) acetylation/methylation (most studied and shown here) and others such as phosphorylation, ubiquitination) can alter chromatin structure from open transcription-permissive to closed transcription-repressive confirmation and *vice versa*. NcRNAs including lncRNAs and microRNAs (miRNAs) can also regulate gene expression at the transcriptional and post-transcriptional levels. K, lysine followed by lysine residue position; R, arginine followed by arginine residue position.

Epigenetics is defined as changes in gene expression, transient or heritable, that are not associated with changes in DNA sequences. The epigenetic status of the genome varies fundamentally in a tissue-specific manner. The overall epigenetic status of a cell is termed ‘epigenome’ that represents the entire structure of the chromatin including covalent modifications. Changes in the epigenome involve myriad enzymatic activities to methylate DNA and modify histones. With the advancement in high-throughput sequencing and novel sample preparation techniques, researchers are now able to map epigenomic modifications including DNA methylation, histone modification, and non-coding RNAs (ncRNAs) with exquisite genome-wide coverage and astonishing accuracy. These developments are propelling the field of epigenetics and revolutionizing our understating of cell biology in human health and disease. The epigenetic control of gene expression is regulated mainly through mechanisms such as DNA methylation, histone modifications and ncRNAs. The cell-specific transcriptional profile is regulated by the physical shape and organization of DNA into chromatin. DNA is organized in the form of chromatin inside the nucleus as a DNA–histone complex. This organization not only facilitates the packaging of the DNA into the nucleus but also regulates gene transcription by structural changes in the chromatin. The chromatin structure is central to epigenetic control of gene transcription. This change in chromatin structure is brought by addition or removal of chemical groups such as acetyl or methyl group from histone tails or DNA itself. These mechanisms are generally referred to as ‘epigenetic mechanisms’ (Figure 2).

## DNA methylation

DNA methylation is an enzymatic process of addition of methyl group to cytosine residues at CpG dinucleotides in the genome [28]. A number of DNA methyl-transferases are involved in writing this epigenetic mark. DNMT1 is responsible for maintaining previously established DNA methylation by recognizing hemi-methylated CpG and adding methyl group to the previously unmethylated cytosine. On the other hand, DNMT3a and DNMT3b are responsible for the establishment of *de novo* DNA methylation. At regulatory elements, DNA methylation can mediate gene silencing through direct inhibition of the binding of methylation-dependent transcriptional activators [29] or indirectly by altering the affinity of proteins involved in the chromatin remodeling such as MeCP2 that recognize and bind methylated CpG sites to recruit chromatin modifiers [30–32]. DNA methylation does not function in isolation but in fact, there is interplay between DNA methylation and histone modification to form a complex epigenome to regulate genome expression.

## Histone modifications

DNA is packaged by histone into chromatin, which is essential for nuclear functions such as transcription, DNA replication and repair [33]. The canonical repeating unit of the chromatin fiber, the nucleosome is composed of approximately 147 bp of double-stranded DNA wrapped around a core of eight histone protein molecules (two copies of each H2A, H2B, H3 and H4) [34,35]. The unstructured tail extensions of histones in the nucleosomes are chemically modified at lysine, arginine, serine and threonine residue by processes such as acetylation, methylation, phosphorylation, ubiquitination and sumoylation to regulate gene expression (Figure 2) [35]. These post-translational modifications (PTMs) can structurally remodel chromatin to form transcriptionally active euchromatin and inactive heterochromatin [36]. Histone acetylation in general relaxes histone–DNA interactions and is correlated with transcriptional activation of genes whereas deacetylation is associated with chromatin compaction and transcriptional repression [37]. Histone acetylation is the process of enzymatic transfer of an acetyl group from acetyl coenzyme A to the amino group of histone lysine residues [37]. Acetylation of histone lysine removes positive charge on histone tails, thereby decreasing the interaction with negatively charged phosphate group of DNA. Consequently, the condensed chromatin structure is transformed into a more relaxed structure allowing greater access for the transcription machinery to come in contact with the DNA template for gene transcription [37]. Acetylation can occur on lysine (K) residues on histones H3 (K9, K14, K18, K23 and K27) and H4 (K5, K8, K12, K16 and K20) [33,38]. Histone acetylation has been correlated with transcriptional activation of genes by forming a relaxed transcriptionally active chromatin structure, however another implication is to provide a site for transcription factor and adaptor proteins binding. Transcription factor complexes can read PTMs on histones, leading to regulatory changes. Histones are acetylated at lysine residue by histone acetyl-transferases (HATs). HATs function as co-activators of transcription as they cannot bind to DNA directly and need other co-activators for targeted promoter recruitment [39]. The involvement of HATs and histone acetylation in disease has led to the development of pharmacological agents to prevent the irregular patterns in HAT activity.

Histone deacetylation is an enzymatic process of removal of acetyl group, an opposing activity to acetylation, which is mediated by histone deacetylase (HDAC) enzymes. Histone deacetylation is associated with transcriptional repression by promoting the compact chromatin state [40]. HDAC activity is also targeted to other non-histone proteins including the transcription factor p53, nuclear receptors and cell cycle regulating proteins, hence more recently referred as lysine deacetylases (KDACs) [41,42]. The activation or repression of pathways detrimental to the onset of disease such as cancer has led to the development of HDAC inhibitors (HDACi) [43]. HDACi generally activate gene expression following inhibition of HDAC-dependent removal of acetyl groups from histone tails.

Histone lysine methylation, depending on the residue involved, can be associated with active or repressive transcription [44]. For example, histone H3 lysine 4 trimethylation (H3K4me3) is associated with active chromatin, whereas histone H3 lysine 9 trimethylation (H3K9me3) and histone H3 lysine 27 trimethylation (H3K27me3) are associated with transcriptionally inactive chromatin. These different patterns of PTM of histones are read and recognized by various proteins for gene regulation. These reader proteins have specific domains to identify a particular modification such as bromodomain to recognize acetylated histones and chromodomain to recognize methylated histones [45]. However, the epigenetic control of the transcriptional state of a gene is a complex process involving multiple histone and DNA modifications. Importantly, enzyme-mediated epigenetic changes are reversible and amenable to pharmacological intervention and thus represent suitable therapeutic targets.

## NcRNA

The DNA inside the nuclei is the blueprint of life that is transcribed into protein coding and non-protein coding RNA sequences. The majority of DNA encodes for RNA molecules that are not translatable into proteins, instead have other increasingly recognized gene regulatory functions. These RNAs are termed ncRNAs and are grouped into long ncRNAs (transcripts longer than 200 bp) and short ncRNAs (transcripts smaller than 200 bp) such as microRNAs (miRNAs). Both long and short ncRNAs are functionally important in epigenetic regulation of gene expression at transcriptional and post-transcriptional levels in both health and disease.

## Epigenetic changes in endothelial cells in atherosclerosis

The considerable amount of experimental and clinical data suggests the endothelial dysfunction is an early sign of the development of atherosclerosis [46]. Important risk factors that are independently linked to endothelial dysfunction include low shear stress, hyperglycemia, hyperlipidemia and aging, and the endothelial epigenome is shown to respond to these stimuli. Epigenetic mechanisms have been demonstrated to be linked to the oxidative stress and inflammation leading to endothelial dysfunction. Several *in vitro* studies highlighted the critical role of epigenetic mechanisms in endothelial dysfunction reviewed elsewhere [8,10,47]. In this section, we describe the *in vivo* evidence of the dynamic endothelial epigenome in response to two important atherosclerotic risk factors, specifically TBF and hyperglycemia.

Fluid mechanical forces generated by blood flow are known to cause structural, functional and transcriptional changes in the vascular endothelium [48]. The earliest atherosclerotic lesions develop where blood flow is disturbed with relatively low shear stress such as where vessels branch and bend [49]. Shear stress is the tangential force of the flowing blood on the vascular endothelium. Straight parts of arteries that are generally spared from atherosclerotic plaques are exposed to sustained laminar blood flow with high shear stress. In contrast, atherosclerotic plaques develop predominantly at branches, bends and bifurcations in the arterial tree because these sites are exposed to TBF that exerts low shear stress on the vascular wall. The molecular mechanisms connecting cellular responses to flow-mediated hemodynamic forces are poorly understood. Epigenetic mechanisms are emerging as important regulators of hemodynamic-mediated transcriptional changes [50]. For instance, a catalytic component of the Polycomb repressive complex 2 (PRC2) that is responsible for tri-methylation of lysine 27 on histone 3 is up-regulated in atherosusceptible endothelium from TBF regions of blood vessels [51,52]. The PRC2 methylates lysine 27 of histone H3 (H3K27me3) through its catalytic component EZH2 [53]. H3K27me3 is tightly associated with inactive gene promoters suppressing gene expression [53]. Multiple lines of evidence have implicated EZH2 up-regulation in the development and progression of atherosclerosis [54–56]. For instance, a recent study has shown increased H3K27me3 in endothelial cells isolated from human early and advanced atherosclerotic plaque [54] in contrast with a decrease in global H3K27me3 in the whole vessels suggesting endothelial-specific H3K27me3 plays an important role in atherosclerotic lesion development [57,58]. Further evidence for a role for EZH2 in atherosclerosis is provided by its enhanced expression in atherosusceptible endothelium from disturbed blood flow regions of blood vessels [51,52]. Mechanosensitive transcription factors NRF2 and KLF2 that regulate many shear responsive genes have been shown to be regulated by HDACs (HDACs,1, 2, 3, 5 and 7) *in vivo* in rats [59]. Furthermore, HDAC3, 5 and 7 have been identified as mechanosensitive chromatin modifiers that control expression of miR-10a in endothelial cells in shear stress modulation of vascular phenotype and function [60].

*In vivo* studies have also demonstrated the critical role of DNA methylation in regulating flow-mediated transcriptional changes in vascular endothelial cells. Turbulent flow has been shown to increase promoter methylation levels of mechanosensitive genes such as *KLF3*, *KLF4* and *HoxA5*. Promoter hypermethylation was inversely correlated with gene expression of these genes [61,62]. In addition, studies have shown DNMT1-dependent genome-wide hypermethylation in endothelium in partially ligated mouse arteries, which is an *in vivo* model of low shear stress [63,64].

MiRNAs have also been identified as important gene regulators at both transcriptional and post-transcriptional levels in flow-induced endothelial gene expression changes in atherosclerosis [65–67]. The expression of an anti-inflammatory, NFκB signaling pathway targeting miRNA, miR-10a was found down-regulated in endothelial cells in porcine aortic arch, a region of TBF as compared with thoracic aorta. Similarly, another mechanosensitive miRNAs miR-92a was identified in Ldlr^−/−^ mice and in pigs targeting endothelial inflammation [68,69]. Using a partial carotid ligation model of flow-induced atherosclerosis, two mechanosensitive athero-miRNAs miR-712 and miR-205 were identified [70,71]. The expression of these miRNAs was elevated by turbulent flow which was associated with endothelial inflammation. As a nuclear mechanosensitive athero-miR, a recent study has demonstrated that caspase 3 inhibition by nuclear enrichment of miR126-5p confers endothelial integrity in atheroprotected regions of arteries suggesting that epigenetic changes are important factors in endothelial integrity [72].

In patients with diabetes, hyperglycemia induces epigenetic changes triggering the expression of genes associated with endothelial dysfunction [10,73,74]. These early epigenetic changes due to poor metabolic control are in part responsible for the ‘metabolic karma’ phenomenon [75]. The DCCT and EDIC trial (type 1 diabetes) as well as the Steno and UKPDS trials (type 2 diabetes) suggested that previous exposure to high glucose levels has long lasting deleterious effects on cardiovascular outcomes despite subsequent better diabetes control [10,76]. Exposure of the endothelial cell layer to chronic hyperglycemia induces a proatherogenic transcriptional profile, which leads to endothelial dysfunction and atherosclerosis. SET7 is a histone methyl transferase responsible for monomethylation of lysine 4 on histone 3 (H3K4). In endothelial cells, SET7 has been shown to drive glucose-induced inflammation by regulating transcription factor NFκB [11]. In patients with type 2 diabetes (T2D), significant correlation of SET7 expression in peripheral blood mononuclear cells (PBMCs) with oxidative stress marker 8-isoprostaglandin F_2α_ and flow-mediated dilation was observed. This correlational study showed that SET7-driven epigenetic changes contribute to vascular dysfunction in patients with T2D [77]. Furthermore, H3K9 hyperacetylation of multiple genes including *HMOX1, IL-8, HMOX1, MMP10, Cox2* and *TNFα* has been detected in vascular cells and immunocytes in patients with diabetes [78,79].

Given the importance of endothelial dysfunction in atherosclerosis development and the emerging role of the environment-responsive epigenome in atherosclerosis, there is still a major lack of knowledge specifically in *in vivo* settings, which needs to be elucidated in order to understand the mechanisms involved in these early steps of vascular injury.

## Immune cell epigenetics in atherosclerosis

Due to ongoing recruitment, differentiation and local proliferation, monocyte-derived macrophages are the dominant cell population of the innate immune system in the atherosclerotic plaque and play a critical role in the disease development and progression. Macrophages are a crucial contributor to plaque progression through secretion of pro-inflammatory mediators and apoptotic death leading to necrosis. Upon apoptosis, macrophages release tissue factors and lipid contents forming a prothrombotic necrotic core, an important contributor to plaque instability. Inhibition of cholesterol efflux in macrophages causes foam cell formation, a hallmark of plaque development [80]. EZH2 has been shown to promote foam cell formation by targeting ABCA1 thus accelerating atherosclerosis [81]. The HDACs SIRT1 and SIRT 6, on the other hand have been shown to enhance cholesterol efflux by the activation of ABCA1 and ABCG1 blunting formation of foam cells and associated inflammation [82]. Recently, myeloid-specific HDAC inhibition using an esterase-sensitive motif (ESM) technology has been shown to impair maturation and activation of peritoneal macrophages with limited efficacy on atherosclerosis [83]. This technology conjugates ESM on to small molecules for targeting those cells that express Carboxylesterase 1 (CES1), such as mononuclear myeloid cells [83].

Macrophages show remarkable plasticity by adopting to various stimuli in tissue-specific environment. Conventionally, and largely in an *in vitro* paradigm, active macrophages have been characterized into pro-inflammatory M1 and anti-inflammatory M2 phenotypes. However, macrophage polarization *in vivo* is much more complex and has been reviewed elsewhere [84]. Several epigenetic enzymes have been implicated in regulation of classical macrophage phenotypes including histone methyltransferase and demethylase such as EZH2 and Jumanji domain-containing protein D3 (JMJD3) and histone acetyltransferase and deacetylases such as P300, SIRT1, SIRT6, HDAC3 and HDAC9 indicating that histone modification is linked to macrophages activation in atherosclerosis [85]. The histone-modifying enzymes that have been shown to regulate macrophage function toward a M2 phenotypes include HDAC4, SIRT2, JMJD3, PRMT1, SMYD3 whereas HDAC3 and HDAC9 have been shown to oppose M2 polarization. The M1 phenotype has been shown to be positively regulated by several chromatin modifiers including JMJD2D, SET7 UTX, HDAC1 and HDAC3 whereas histone modifiers that inhibit M1 polarization include JMJD1A, SMYD2, SIRT1 and SIRT2 (*in vitro* studies, reviewed in [85]). A study has shown that up-regulation of SMYD3 (SET and MYND domain containing protein 3) favors the M2 phenotype in human monocyte-derived macrophages *in vitro*. SMYD3 is a methyltransferase inducing di and tri-methylation of lysine 4 on histone H3 (H3K4) which is associated with transcriptional activation. Elevated expression of SMYD3 was associated with increased levels of trimethylation of H3K4 at a lipoxygenase M2 marker, *ALOX-15* promoter mediating its transcriptional activation [86]. JMJD3 is a histone demethylase that removes the repressive trimethylation marks on lysine 27 of histone H3 facilitating the gene transcription. JMJD3 has been shown to be involved in the regulation of pro-fibrotic gene expression via H3K27 demethylation in macrophage-derived foam cells. RNA sequencing of peritoneal foam cells in myeloid cell-specific jmjd3 deficient mice showed that JMJD3 is involved in pro-fibrotic signature of macrophage-derived foam cells *in vivo* [87].

**Figure 3:**
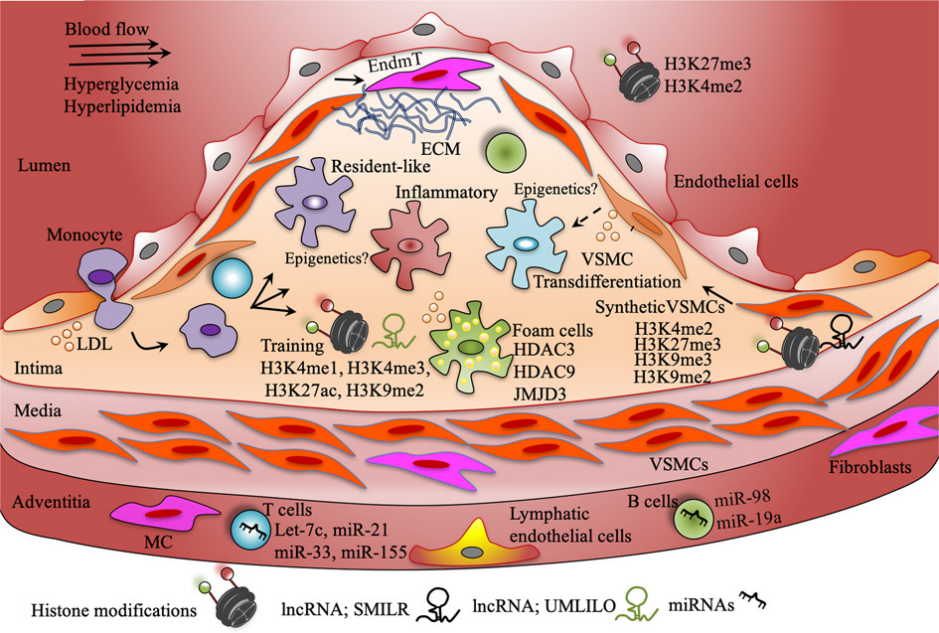
Epigenetics in vascular cells at play in the atherosclerotic plaque development Atherosclerotic plaque development is a complex process. Histone modifications have been shown to play important roles in endothelial dysfunction. Histone marks and ncRNAs have been demonstrated to be involved in trained innate immunity. Several miRNAs have been shown to regulate functions of adoptive immune cells such as T and B lymphocytes in atherosclerosis. Recent single cell sequencing studies have identified at least three macrophage subtypes (differently colored here). Epigenetic mechanisms need to be investigated in these subpopulations. However, histone deacetylation and demethylation have been shown to be associated with foam cell formation. Recent evidence indicates VSMCs oligoclonal expansion and transdifferentiation into macrophage-like cells. Several histone marks have been identified *in vivo* associated with the novel role of VSMCs in atherosclerosis. H3, histone 3; K4, lysine 4; K9, lysine 9; K27, lysine 27; me1, mono-methylation; me2, di-methylation; me3, tri-methylation; EndmT, endothelial-to-mesenchymal transition.

Macrophage activation with different stimuli results in different phenotype. For example, IL4/IL13 stimulation results in anti-inflammatory macrophages, LPS/INFγ stimulation leads to an inflammatory phenotype and modified lipid uptake results in foamy macrophages. Multiple epigenetic enzymes have been described as important regulators of these phenotypic changes. For example, JMJD3 has been shown to be involved in anti-inflammatory and inflammatory macrophage phenotypes, whereas HDAC3 and HDAC9 are linked to foamy macrophage and inflammatory macrophage phenotype. Recent evidence indicates a wider range of phenotypic adaptation of macrophages in response to local tissue cues. Several scRNA-seq studies have identified multiple macrophage populations with three prominent macrophage clusters namely resident-like macrophages, inflammatory macrophages and *Trem2*^hi^ foamy macrophages in human and murine atherosclerosis (Figure 3). Epigenetic mechanisms are highly likely to play an important role in macrophage heterogeneity and plasticity identified by scRNA-seq that needs further investigation with the advent of state-of-the-art single cell epigenomic technologies.

Furthermore, until recently, monocytes and macrophages were regarded as immune cells of the less sophisticated innate immune system because they lack the antigen-specific lifelong immunological memory that exist in cells of the adoptive immune system such as B and T lymphocytes. However, recent evidences suggest a specialized immune memory exist in these cells, a phenomenon termed as ‘trained immunity’. Trained immunity refers to the ability of cells of the innate immune system to remember invading agents and respond with augmented strength to reinfection through epigenetic and metabolic rewiring. Importantly, trained immunity is non-specific and long-lasting. It responds strongly to secondary similar or unrelated stimuli and sustains the activated phenotype for a longer period. Various cues including exogenous and endogenous stimuli can induce trained immunity, however our current mechanistic understanding of innate immune memory is mainly based on studies using BCG vaccine (Bacille Calmette-Guerin, a live attenuated vaccine against tuberculosis), β-glucan (components of the cell wall of *Candida albicans*) and oxLDL. This prolonged hyperactive innate immune response has recently been hypothesized as a potential mechanism for non-resolving chronic inflammation in atherosclerosis. Epigenetic remodeling is thought to be at the core of molecular mechanisms associated with trained immunity. Chromatin-based studies have identified associations of specific histone modifications such as H3K4me3, H3K4me1, H3K27ac and H3K9me2 with trained immunity. Induction of trained immunity by BCG vaccination in healthy volunteers was associated with elevated H3K4me3 at the prompters of IL-6 and TNF-α. β-glucan has also been shown to stimulate trained immunity by production of pro-inflammatory cytokines regulated through histone modification, H3K4me3. Genome-wide increase in H3K27ac both at enhancers and promoters has been observed in β-glucan induced innate immune memory in differentiated macrophages [88]. The ATF7 and H3K9 methyltransferase G9a axis has been shown to be linked to a decline in repressive histone modification H3K9me2 on inflammatory genes upon β-glucan or LPS stimulation of murine macrophages [89]. Induction of trained immunity was shown to blunt KDM5 demethylase activity linked to elevated H3K4me3 at trained immunity genes activating transcription in monocytes [90]. More recently, histone methyltransferase SET7, an H3K4me1 writing enzyme was shown to induce trained immunity genes by modifying chromatin accessibility at enhancer regions of genes in primary human monocytes [91].

Enrichment of the transcription-activating histone mark, H3K4me3 occurs at immune-related gene promoters during trained immunity. Recently, β-glucan was shown to epigenetically reprogram immune genes in a long ncRNA-dependent manner in mouse macrophages [92]. Long ncRNA UMLILO regulated chromatin structure at the promoters of immune genes during β-glucan-induced trained immunity. Three-dimensional chromatin looping positioned the UMLILO in the genomic location proximal to essential trained immune gene promoters such as IL-6 and IL1β, facilitating H3K4me3 enrichment by targeting WDR5–MLL1 (WD repeat-containing protein 5 (WDR5)-mixed lineage leukemia protein 1 (MLL1)) complex at these gene promoters.

Antigen-responsive adoptive immune activity contributes significantly to the atherogenesis process. Studies of lymphocyte deficiency in atherosclerotic mouse models such as ApoE^−/−^ and LdlR^−/−^ demonstrated a significant involvement of the adaptive immune system in the atherosclerotic plaque development and progression [93]. Different subsets of T and B lymphocytes show variable pro- and anti-inflammatory response in atherosclerosis. T lymphocytes in the plaques are antigen-experienced memory T cells. Naïve T cells are primed in secondary lymphoid organs by antigen-presenting cells (APCs) such as dendritic cells (DCs). Second activation of these primed T cells occurs in non-lymphoid tissues by APCs. CD4^+^ T cells are abundant, although CD8^+^ cells are also found in the plaque. CD4^+^ T helper type 1 (T_H_1) cells are considered pro-atherogenic by promoting macrophage activation and inflammation through interferon-γ (IFNγ) production whereas regulatory T cells (T_regs_) have atheroprotective functions by limiting the pro-inflammatory response of T_H_ cell subtypes. A reduced number of T_regs_ in peripheral blood was found to be associated with acute coronary disease in atherosclerotic patients [94]. DNA hypermethylation of the *FOXP3* gene was suggested to suppress its expression thereby down-regulating T_regs_ and increasing the risk of acute coronary disease. However, T_regs_ have also been shown to convert into a pro-inflammatory T-cell subtype and become pro-atherogenic during atherosclerosis progression. The role of other T-cell subtypes including T_H_2, T_H_9 and T_H_22 are less investigated. Several miRNAs have been shown to play a critical role in regulating T-cell proliferation and differentiation in atherosclerosis. OxLDL-induced miRNA let-7c expression was shown to be down-regulated by Statin treatment in human naïve T cells isolated from carotid endarterectomy samples from patients co-cultured with pre-treated DCs [95]. Down-regulation of miR let-7c induced T_regs_ was associated with elevated levels of anti-inflammatory cytokine IL-10 [95]. T_regs_ have also been shown to be negatively regulated by miR-21 in coronary heart patients [96]. Inhibition of another miRNAs, miR-33 has also been implicated in T_reg_ differentiation in atherosclerotic mice [97]. Moreover, a recent study revealed a role of an miRNA, miR-155 in CD4^+^ T lymphocyte-mediated immune response in human primary cells *ex vivo* [98]. Endothelial, VSMCs and T cells were used from the same donor to avoid potential immune rejection. Inhibition of miR-155 in naïve CD4^+^ T cells was achieved with lentiviral transfection. These cells were activated by incubating with oxidized LDL-stimulated DCs. The T_H_2 and T_reg_ T lymphocyte population was increased with elevated levels of anti-inflammatory cytokines. Co-culture experiments showed that miR-155 inhibition blunted CD4^+^ T cells to induce endothelial cell apoptosis and to promote VSMCs growth [98]. However, further research is warranted to identify gene targets of miR-155 in CD4^+^ T cells and to validate results of this study in the *in vivo* setting.

Epigenetic changes in concert with transcription factors play crucial roles in B lymphocyte development and differentiation, thereby modulating humoral responses to antigens. Studies investigating epigenetic mechanisms in B cell differentiation in atherosclerosis are scarce. However, several miRNAs have been identified as important regulators of B cell functions in atherosclerosis. The immune regulatory cytokine IL-10 is down-regulated in peripheral B cells in patients with atherosclerosis which was inversely correlated with the expression of miR-19a [99]. Furthermore, TNF-α, INF-γ or IL-4 was shown to repress IL-10 in cultured B cells via increased expression of miR-19a [99]. The anti-inflammatory cytokine IL-10 is also shown to be targeted by another miRNA, miR-98 in peripheral B lymphocytes in patients with coronary artery atherosclerosis [100]. In that study, level of serum cortisol, a psychological stress hormone was higher in patients and inversely correlated with peripheral B-cell frequency. Interestingly, cortisol suppressed IL-10 expression via miR-98 in cultured B lymphocytes suggesting therapeutic potential of targeting miR-98 in atherosclerosis. B-cell subtypes have also contrasting effects on atherosclerosis progression. B2 B lymphocyte depletion ameliorated atherosclerosis in ApoE^−/−^ mice whereas its adoptive transfer aggravated atherosclerosis in lymphocyte deficient ApoE^−/−^ Rag-2^−/−^ mice [101]. In contrast, B1a B cells have been shown to be atheroprotective when B1a cells were selectively transferred to splenectomized ApoE^−/−^ mice, which restored secreting levels of natural IgM antibodies [102]. Future studies targeted to identify epigenetic mechanisms linked to these contrasting roles of the B cell subtypes may identify potential novel therapeutic targets in atherosclerosis.

## Epigenetics of VSMCs in atherosclerosis

Studies have shown that atherosclerosis progression involves cross-talk between immunocytes with endothelial cells and VSMCs. Smooth muscle cells are the major cell population in the vascular wall mainly located in media. In healthy vessels, VSMCs express contractile proteins and function to regulate blood flow and pressure through vascular contraction and relaxation. In response to vascular injury, VSMCs undergo a phenotypic modulation and convert into proliferative synthetic cells that through de-differentiation, migration and proliferation play an important role in atherosclerotic plaque development and progression [103]. VSMCs in the intima were traditionally considered as beneficial as early evidence suggested a plaque stability function attributed to matrix producing VSMCs in advanced atherosclerotic plaques by fibrous cap formation [104–106]. However, more recent evidences indicate the remarkable plasticity of VSMCs suggesting a more complex role for VSMCs contributing to plaque vulnerability. Several recent lineage-tracing studies and scRNA-seq studies revealed the VSMCs heterogeneity and proliferation in a clonal fashion [25,107,108]. These studies have shown that VSMCs can adopt to a less differentiated phenotype that lacks classical markers of contractile VSMCs and may directly promote atherosclerosis.

In response to vascular injury, VSMCs undergo reversible phenotypic transition from contractile to synthetic state. However, studies using *in vivo* genetic fate mapping as clonality tracking systems have provided clear evidence that VSMCs within the atherosclerotic plaque originate from a limited number of clones [15,108–112]. In addition, within the plaque, VSMCs can alter their gene expression profile to resemble various other cell types such as macrophages, mesenchymal stem-like cells, osteochondrocytes and myofibroblasts [14,15,108,113]. These distinct cell types that have a VSMC-origin that may be involved in multiple processes including plaque stability, lesion growth, lipid retention and inflammation. Collectively, the latest consensus is that in response to vascular injury, VSMCs populate injured vessels by expansion in clonal fashion and undergo multiple phenotypic modulations by expressing markers of alternative cell types. Thus, VSMC-derived cells can influence plaque stability as well as vulnerability.

Studies have identified several transcription factors and promoter elements that control the transcription of genes associated with VSMC phenotypic transition including contractile genes. Gene encoding contractile proteins including *ACTA2, CNN1, SM22 and MYH11* are regulated by CC(A/T-rich)_6_GG (CArG) *cis*-regulating elements in their promoter [104]. These elements are recognized and bound by the transcription factor Serum Responsive Factor (SRF) [114]. SRF associates with Myocardin (MYOCD) which is only expressed in the vasculature by VSMCs to achieve cell type-specific expression of CArG-dependent contractile genes [114,115]. Several studies have provided compelling evidence that multiple epigenetic changes are associated with VSMCs phenotypic transition in response to vascular injury *in vivo*.

Reduced levels of gene repressive histone modification, H3K27me3 was observed in medial regions of advanced human peri-renal aortic atherosclerotic plaques by immunohistochemistry staining [116]. Interestingly, no change in expression of the corresponding histone methyltransferase, EZH2 and demethylase, JMJD3 was observed. An early study identified down-regulation of H3K9me3-especific methyltransferase Suv39h1 and reduced levels of the repressive H3K9me3 on inflammatory genes responsible for VSMCs-mediated inflammation in diabetic db/db mice [117]. This study demonstrated that H3K9me3 plays an important role in VSMCs inflammation in diabetes. In addition, reduced levels of H3K9me2 and increased expression of histone demethylase KDM3a were observed in VSMCs of diabetic rats suggesting H3K9me2 may have a role in vascular complications of diabetes [118]. Interestingly, another study identified reduced levels of H3K9me2 and increased levels of H3K4me2 in VSMCs in advanced human atherosclerotic carotid plaques as compared with early plaques by immunohistochemistry [119]. More recently, a study has shown an atheroprotective role for this repressive histone mark, H3K9me2 through regulating a pro-inflammatory response of VSMCs in atherosclerosis [120]. Using a high fat-fed VSMC-lineage tracing transgenic ApoE^−/−^ mouse model, the authors demonstrated that protein levels of H3K9me2 in VSMCs were decreased in atherosclerosis both in the media and in the lesion itself. ChIP analysis in primary human and murine VSMCs identified H3K9me2 enrichment at promoters of important components of VSMCs-mediated inflammation including matrix metalloproteinases MMP3, MMP9, MMP12 and pro-inflammatory cytokine IL-6. Inhibition of H3K9me2 writing enzymes G9A and GLP elevated VSMCs-mediated inflammation *in vitro* and *in vivo*. Furthermore, H3K9me2 enrichment prevented NFκB and cJUN transcription factors binding to the promoters of IL-6 and MMP3 and blunted the inflammatory response in VSMCs. In contrast, G9A and GLP inhibition caused NFκB and cJUN enrichment on IL-6 and MMP3 promoters as a result of decreased H3K9me2 augmenting VSMCs inflammation [120]. Gene repressing activity of histone deacetylation by HDACs have also been studied in VSMCs phenotypic modulation. A study has shown KLF4-dependent binding of pELk and HDAC2 at CArG element containing VSMC marker genes such as *Tagln* blunting their expression after ligation-induced carotid injury *in vivo* [121].

DNA methylation may also play a crucial role in VSMCs phenotypic transition, however reports of direct evidence of DNA methylation associated with VSMCs phenotypic modulation in atherosclerosis *in vivo* are limited. The DNA demethylase Ten Eleven Translocation 2 (TET2) have been shown to regulate the expression of SRF and contractile genes such as *MYOCD* and *MYH11* in human VSMCs. Expression of TET2 is inversely correlated with severity of atherosclerosis in patients and its knockdown in mouse exacerbates vascular response to injury [122]. Moreover, a recent study demonstrated that DNA methylation modulates expression of an miRNA, miR-128-3p that plays a critical role in VSMC phenotypic transition in vascular injury [123].

Recent evidence suggests that ncRNAs including long ncRNA and miRNAs also play a role in VSMCs phenotypic transition. Smooth muscle-induced lncRNA enhances replication (SMILR; Ensembl: RP11-94A24.1) was found to be up-regulated in human unstable atherosclerotic plaques. Upon stimulation with inflammatory mediators (platelet-derived growth factor (PDGF) and IL-1α), Primary human saphenous-derived VSMCs (HSVSMCs) showed increased SMILR expression linked to inflammation and VSMC proliferation [124]. The miR143/145 cluster regulates VSMC specific gene expression controlling plasticity and contractility. MiRNAs miR-143 and miR-145 deficiency showed reduced atherosclerosis in LdlR^−/−^ mice [125]. Furthermore, these miRNAs have been shown responsible for aortic smooth muscle cells transition to dysfunctional macrophage-like cells in response to lipid loading [126]. A recent study identified a novel miRNA, miR-128-3p as a crucial regulator of VSMC phenotypic modulation. Stimulation with a potent atherogenic agent PDFG, down-regulated miR128-3p expression inducing VSMC differentiation. Interestingly, aortic expression of miR128-3p was decreased in ApoE^−/−^ mice fed a high cholesterol diet. Furthermore, gain and loss-of-function *in vivo* and *in vitro* experiments showed that miR128-3p regulates VSMC phenotypic transition via an miR-128-3p/KLF4-MYH11 axis. KLF4 was identified as a direct target of miR128-3p and KLF4 then regulates MYH11 through positive modulation of promoter DNA methylation maintaining the differentiated state [123].

These epigenetic targets including miRNAs represent attractive potential candidates to preserve plaque stabilizing functions of VSMCs and to limit detrimental SMC phenotypic behavior in vascular pathologies.

## Fibroblast epigenetics in atherosclerosis

The outer most layer of vessel wall is termed as adventitia that is surrounded by PVAT. The adventitia contains several types of cells including fibroblasts, VSMCs, adipocytes, pericytes and resident immune cells including macrophages, DCs, mast cells, T cells and B cells [127]. Fibroblasts are the most common cells in the adventitia of a healthy vessel that produce extracellular matrix fibers including collagen type I and III, proteoglycans and fibronectin [128]. The activated fibroblast cells express α smooth muscle actin (α-SMA) and are termed as myofibroblasts. Upon activation, adventitial fibroblasts have been shown to infiltrate atherosclerotic lesions and contribute to neointima and fibrous cap formation [108,128–130]. However, new insights from single cell sequencing indicate the plastic and heterogenic nature of fibroblasts in healthy and atherosclerotic vessels [16]. Interestingly, scRNA-seq analysis of the healthy murine aorta showed that fibroblasts were the second most abundant (∼33%) cells after VSMCs (∼40%) even after removal of PVAT indicating existence of plastic and heterogenic fibroblasts in healthy vessel wall [24]. Two fibroblasts subpopulation were identified by scRNA-seq in human and murine atherosclerotic vessels further imposing the heterogenic nature of these cells [24]. Other studies have identified additional fibroblast subpopulations [108,131]. Recent studies have revealed several origins of atherosclerosis-associated fibroblasts including VSMC, endothelial cells and adventitial stem/progenitor cells attributed to their plasticity and heterogeneity nature [128,132]. In addition, scRNA-seq studies have shown phenotypic modulation of fibroblasts into other cell types and vice versa in atherosclerosis [131]. This phenotypic modulation is consistent with changes in gene expression profile of these fibroblasts’ subpopulations in atherosclerosis. Epigenetic mechanisms are highly likely to be involved in regulating fibroblast phenotypic transition in atherosclerosis. Several studies have unravelled the role of multiple epigenetics mechanisms in myofibroblasts differentiation. These studies have identified that the DNA methyltransferases, HATs, HDACs and miRNAs including miR-21, miR-29, miR-132 and miR-155 associated with myofibroblast differentiation [17,133–135]. However, studies investigating these epigenetic changes directly in atherosclerotic vessels are scarce. This could be due to the limited number of well-defined fibroblasts in atherosclerotic lesions that are required for epigenetic analysis. Combining lineage tracing experiments with scRNA-seq may advance our understanding of the role of epigenetic changes in fibroblasts functions in atherosclerosis.

## Epigenetic therapies for atherosclerosis

Although LDL oxidation remains a major culprit in the atherosclerotic process, studies appearing in rapid succession are supporting the pivotal role of vascular inflammation and immune dysregulation as key drivers of atherosclerosis [136]. Targeting epigenetic pathways controlling vascular inflammatory response represents indeed a new frontier in pharmaceutical research and might contribute to reduce the burden of vascular disease in this setting [137]. Over the last years, advances in epigenetic research have a led to a deep comprehension of chromatin dynamics and DNA methylation thus paving the way for the development of several chromatin modifying drugs. Many of these compounds modulate the activity of enzymes involved in DNA methylation or post-translational histone modifications thus affecting transcriptional programs implicated in atherosclerotic vascular disease [138]. The general limitation of epigenetic therapy is that compounds targeting epigenetic modifiers have broader effects. However, technical advances facilitating specific epigenetic editing may provide solution to address this limitation [83].

Dietary compounds such as folic acid are potent modulators of chromatin structure and can be considered promising epigenetic drugs in patients with cardiovascular disease [139]. The methyl-group responsible for DNA and histone methylation originates from folate metabolism. In this process, folic acid is first reduced to dihydrofolate by dihydrofolate reductase and subsequently to tetrahydrofolate (THF), which after several steps is reduced to 5-methylTHF by the methylenetetrahydrofolate reductase (MTHFR) enzyme [140]. Folate-enriched diets have shown to modulate DNA methylation and to affect obesity and hypertension-induced endothelial dysfunction, liver steatosis (via modulation of PPARα signaling) and adipogenesis [139,141]. Epigenetic remodeling induced by folate rescues obesity-related pro-inflammatory transcriptional signatures and, interestingly enough, folate-induced chromatin modifications are transmitted across multiple generations in rats [142]. The latter aspect highlights the notion that epigenetic therapies may exert protective effects which extend to the progeny and further generations. Restoration of CpG methylation by folates prevents the up-regulation of the mitochondrial adaptor p66^Shc^, a pivotal regulator of vascular oxidative stress, endothelial dysfunction and atherosclerosis [143–145]. The clinical relevance of this observation is supported by the notion that CpG methylation of p66^Shc^ promoter is significantly reduced in peripheral blood leukocytes from patients with coronary artery disease and high plasma homocysteine levels [143]. Vitamin B6, vitamin B12, betaine and its precursor choline are dietary compounds also involved in the regulation of DNA methylation in atherosclerosis. For example, betaine supplementation in apolipoprotein ApoE deficient mice was able to prevent atherosclerotic lesion formation and growth [146]. Along the same line, sulforaphane (SFN), an organosulfur compound found in broccoli sprouts, and epigallocatechin-3-gallate (EGCG), the most abundant catechin in green tea, have also shown to modulate vascular oxidative stress and inflammation [147]. SFN administration prevents pulmonary vascular remodeling, inflammation and fibrosis via inhibition of the Nrf2 pathway [148]. Moreover, SFN suppresses endothelial inflammation by preventing TNF-α-mediated secretion of VCAM-1, ICAM-1, E-selectin, Endothelin-1 as well as hyperglycemia-induced endothelial dysfunction [149]. EGCG also exerts anti-inflammatory effects. In cultured endothelial cells, the compound was recently found to attenuate LPS-induced ICAM-1 up-regulation and to accelerate re-endothelialization in the diabetic vasculature via the Akt/eNOS pathway [150,151]. Of note, low doses of EGCG prevented atherosclerosis in LDL receptor knockout mice [152]. Given the pivotal role of DNA CpG methylation in the pathogenesis of atherosclerosis, over the last few years several studies investigated the potential relevance of methylation-editing interventions [140]. Treatment of VSMCs with 5-azacytidine, a compound able to inhibit DNA methyltransferase activity, resulted in reduced methylation of eNOS promoter and subsequent gene up-regulation [153]. Moreover, 5-AZA administration was found to restore the CpG methylation pattern at the promoter of mechanosensitive and inflammatory genes, thus preventing vascular remodeling and endothelial dysfunction [154]. Similarly, to what observed with 5-AZA, treatment with DNMT inhibitors induced demethylation of the eNOS promoter thus favoring its transcription in vascular cells [155,156]. Given the impairment of NO signaling in atherosclerosis, epigenetic-based approaches for the modulation of eNOS expression in this setting would represent an attractive opportunity. Beside DNA methylation, pharmacological targeting of histone modifications is also a potential epigenetic intervention to combat features of atherosclerotic vascular disease [157]. The HDACi Vorinostat prevents eNOS uncoupling and NF-kB transcriptional programs in the diabetic vasculature [158], and has shown to reactivate autophagic flux in the heart [159,160]. These findings are of potential relevance as defective autophagy is emerging as an important underpinning of endothelial dysfunction and atherosclerosis in obesity [161]. Potent anti-inflammatory effects were also observed with the HDACi sodium butyrate, which was shown to suppress NF-kB signaling and NF-kB-dependent inflammatory molecules (i.e. TNF-α, IL-6, VCAM-1 and ICAM-1) in human endothelial cells and circulating peripheral blood mononuclear cells [162,163]. Chronic treatment with butyrate also prevents metabolic alterations in experimental models of obesity and diabetes by enhancing oxidative phosphorylation and β-oxidation in mitochondria [164]. Butyrate-dependent effects on mitochondrial metabolism are mainly due to changes in chromatin accessibility and miRNA expression (i.e. miR-133a-3p) leading to transcriptional modulation of PGC-1α [156]. Moreover, Trichostatin A (TSA), an organic compound which selectively inhibits the class I and II HDAC, was found to suppress the transcription of TNF-α, the latter being a detrimental signature involved in the ET-1/NO system imbalance, endothelial dysfunction and atherosclerosis [165,166]. Inhibition of the histone deacetylase SIRT1 by resveratrol has shown to attenuate inflammation, oxidative stress, and to rescue endothelial dysfunction in experimental models and patients with cardiometabolic disturbances [167–169]. These effects on vascular function were driven by inhibition of TNFα-induced activation of NAD(P)H oxidase and preservation of eNOS activity [170,171]. However, the beneficial effects of resveratrol were not confirmed by other studies suggesting that more research is needed in this area [172]. Pharmacological activation of SIRT3 has also demonstrated to prevent accumulation of mitochondrial ROS mainly via histone deacetylation and subsequent increase in MnSOD transcription [173]. Beside HDACs, emerging evidence indicates that therapeutic targeting of acetyltransferases (HATs) contributes to maintain vascular homeostasis by controlling transcriptional programs implicated in antioxidant and redox signaling [174]. A clear example is represented by curcumin, an important bioactive component of turmeric that has been widely applied as a traditional medicine to prevent and treat various diseases [175]. This drug acts by inducing a proteasome-dependent degradation of the HAT p300 and the closely related CBP protein, thus reducing chromatin accessibility on the promoter of genes regulating cellular growth, proliferation, survival, inflammation and oxidative stress [174]. Of interest, chronic supplementation with the HAT inhibitor Curcumin improves vascular endothelial function in healthy middle-aged and older adults by increasing NO bioavailability and reducing oxidative stress [176].

Pharmacological modulation of epigenetic reader proteins, namely BETs (bromodomain and extraterminal-containing protein family) is gaining increasing attention. BETs—which include BRD2, BRD3, and BRD4 and the testis-restricted BRDT—are epigenetic reader proteins that bind to specific acetylated lysine residues on histone tails where they facilitate the assembly of transcription complexes including transcription factors and transcriptional machinery like RNA Polymerase II [157]. Recent evidence indicates that BETs induce specific transcriptional programs in endothelial cells, VSMCs and inflammatory cells [157]. BET inhibition, including the use of specific chemical BET inhibitors like JQ-1, has shown to attenuate atherosclerosis and intimal hyperplasia in experimental models [157]. These beneficial effects are explained by suppression of vascular inflammation as well as by lipid-lowering effects. Indeed, the BET inhibitor apabetalone (RVX-208) stimulates reverse cholesterol transport both *in vitro* and *in vivo* by inducing ApoAI expression and increasing HDL levels [177,178]. Notably, this drug prevents hyperglycemia-induced up-regulation of IL-1β, IL-6 and TNF-α in human endothelial cells and in aortic plaques from ApoE^−/−^ mice [179]. Apabetalone has also shown to decrease systemic inflammation in humans, as assessed by C-reactive protein levels [179]. Of clinical relevance, a pooled analysis of non-randomized studies showed that treatment with apabetalone was associated with fewer cardiovascular events as compared with placebo [180]. The recent phase III BETonMACE trial, designed to investigate the impact of apabetalone on cardiovascular outcomes in 2425 patients with diabetes after an acute coronary syndrome, failed to meet the primary cardiovascular endpoint of cardiovascular death, non-fatal myocardial infarction, or stroke [181]. However, the drug showed a highly favorable profile on secondary endpoints, namely heart failure. Therefore, despite the negative results of BETonMACE trial, there is still potential for future clinical trials targeting BET proteins in CVD in specific subsets of patients. Future studies with apabetalone or other BET inhibitors should be performed in larger cohorts, and possibly target more specific patient groups such as in patients with established CVD and an inflammatory risk profile.

## Concluding remarks

The cellular composition of atherosclerotic plaque is central to plaque stability. The atherosclerotic plaque represents a highly complex tissue with multiple cell types each contributing in their own way at different stages of disease progression. The phenotypic modulation of different vascular cell types during plaque development is a consequence of changes in gene expression. This phenotypic modulation is a cellular response to detrimental environmental stimuli. The underlying transcription-controlling epigenetic changes are reversible and pharmacologically modifiable. Indeed, it is crucial to understand cell-specific epigenetic modifications that through gene expression changes contribute to cell-specific functions within this complex plaque development process. Drugs targeting epigenetic modifiers have global effects, a general limitation of epigenetic therapy, however, a recent study has shown that macrophage-specific HDACi delivery reduced atherosclerotic plaque size raising hope for specific epigenetic editing and their functional consequnces [83]. Advances in techniques targeting specific cell types and gene loci hold a great promise in addressing these limitations. Furthermore, the latest single cell technological advances hold great potential to identify cell type-specific novel epigenetic targets.

## Open Access

Open access for this article was enabled by the participation of Monash University in an all-inclusive *Read & Publish* pilot with Portland Press and the Biochemical Society under a transformative agreement with CAUL.

## Abbreviations

5-AZA: 5-Azacytidine
ABCA1: ATP binding cassette subfamily A member 1
ABCG1: ATP binding cassette subfamily G member 1
APC: antigen-presenting cell
BCG: Bacille Calmette-Guerin
BET: bromodomain and extraterminal-containing protein family
CArG: CC(A/T-rich)GG
ChIP: chromatin immunoprecipitation
CyTOF: cytometry time of flight
DC: dendritic cell
DNMT: DNA methyltransferase
EGCG: epigallocatechin-3-gallate
eNOS: endothelial nitric oxide synthase
ESM: esterase-sensitive motif
ET-1: endothelin 1
EZH2: enhancer of zeste homolog 2
FACS: fluorescence-assisted cell sorting
HAT: histone acetyl-transferase
HDAC: histone deacetylase
HDACi: HDAC inhibitor
H3K4: lysine 4 on histone 3
H3K4me3: histone H3 lysine 4 trimethylation
H3K9me3: histone H3 lysine 9 trimethylation
H3K27me3: histone H3 lysine 27 trimethylation
IL: interleukin
INF: interferon
JMJD3: Jumanji domain-containing protein D3
LDL: low-density lipoprotein
miRNA: microRNA
MYOCD: Myocardin
ncRNA: non-coding RNA
NO: nitric oxide
oxLDL: oxidized low-density lipoprotein
PRC2: Polycomb repressive complex 2
PTM: post-translational modification
PVAT: perivascular adipose tissue
scRNA-seq: single-cell RNA sequencing
SFN: sulforaphane
SIRT: Sirtuin
SMILR: smooth muscle-induced lncRNA enhances replication
SMYD3: SET and MYND domain containing protein 3
SRF: Serum Responsive Factor
TBF: turbulent blood flow
TET2: Ten Eleven Translocation 2
THF: tetrahydrofolate
T_reg_: regulatory T cell
VSMC: vascular smooth muscle cell
